# Human rotavirus vaccine *Rotarix*™ provides protection against diverse circulating rotavirus strains in African infants: a randomized controlled trial

**DOI:** 10.1186/1471-2334-12-213

**Published:** 2012-09-13

**Authors:** Andrew Duncan Steele, Kathleen M Neuzil, Nigel A Cunliffe, Shabir A Madhi, Pieter Bos, Bagrey Ngwira, Desiree Witte, Stacy Todd, Cheryl Louw, Mari Kirsten, Sanet Aspinall, Leen Jan Van Doorn, Alain Bouckenooghe, Pemmaraju V Suryakiran, Htay Htay Han

**Affiliations:** 1 Rotavirus Vaccine Program, PATH, 2201 Westlake Ave, Seattle, WA, 98121, USA; 2 Initiative for Vaccine Research, WHO, 22 Appia Ave, Geneva, 1211, Switzerland; 3 Department of Clinical Infection, Microbiology & Immunology, Institute of Infection and Global Health, University of Liverpool, Liverpool, England; 4 National Institute for Communicable Diseases: a division of National Health Laboratory Services, Sandringham, South Africa; 5 Department of Science and Technology National Research Foundation: Vaccine Preventable Diseases, University of the Witwatersrand, Johannesburg, South Africa; 6 MRC Diarrhoeal Pathogens Research Unit, University of Limpopo, Limpopo, South Africa; 7 Department of Community Health, College of Medicine, Blantyre, Malawi; 8 Madibeng Centre for Research, Brits, South Africa; 9 Department of Paediatric Surgery, University of Pretoria, Pretoria, RSA; 10 Synexus Clinical Research SA/Rota Consortium, Pretoria, South Africa; 11 DDL Diagnostic Laboratory, Voorburg, The Netherlands; 12 Sanofi Pasteur, Singapore, Singapore; 13 GSK Biologicals, Rixensart, Belgium

## Abstract

**Background:**

Rotaviruses are the most important cause of severe acute gastroenteritis worldwide in children <5 years of age. The human, G1P[8] rotavirus vaccine *Rotarix*™ significantly reduced severe rotavirus gastroenteritis episodes in a Phase III clinical trial conducted in infants in South Africa and Malawi. This paper examines rotavirus vaccine efficacy in preventing severe rotavirus gastroenteritis, during infancy, caused by the various G and P rotavirus types encountered during the first rotavirus-season.

**Methods:**

Healthy infants aged 5–10 weeks were enrolled and randomized into three groups to receive either two (10 and 14 weeks) or three doses of *Rotarix*™ (together forming the pooled *Rotarix™* group) or three doses of placebo at a 6,10,14-week schedule. Weekly home visits were conducted to identify gastroenteritis episodes. Rotaviruses were detected by ELISA and genotyped by RT-PCR and nucleotide sequencing. The percentage of infants with severe rotavirus gastroenteritis caused by the circulating G and P types from 2 weeks post-last dose until one year of age and the corresponding vaccine efficacy was calculated with 95% CI.

**Results:**

Overall, 4939 infants were vaccinated and 4417 (pooled *Rotarix™* = 2974; placebo = 1443) were included in the per protocol efficacy cohort. G1 wild-type was detected in 23 (1.6%) severe rotavirus gastroenteritis episodes from the placebo group. This was followed in order of detection by G12 (15 [1%] in placebo) and G8 types (15 [1%] in placebo). Vaccine efficacy against G1 wild-type, G12 and G8 types were 64.1% (95% CI: 29.9%; 82%), 51.5% (95% CI:-6.5%; 77.9%) and 64.4% (95% CI: 17.1%; 85.2%), respectively. Genotype P[8] was the predominant circulating P type and was detected in 38 (2.6%) severe rotavirus gastroenteritis cases in placebo group. The remaining circulating P types comprised of P[4] (20 [1.4%] in placebo) and P[6] (13 [0.9%] in placebo). Vaccine efficacy against P[8] was 59.1% (95% CI: 32.8%; 75.3%), P[4] was 70.9% (95% CI: 37.5%; 87.0%) and P[6] was 55.2% (95% CI: -6.5%; 81.3%)

**Conclusions:**

*Rotarix*™ vaccine demonstrated efficacy against severe gastroenteritis caused by diverse circulating rotavirus types. These data add to a growing body of evidence supporting heterotypic protection provided by *Rotarix™*.

**Trial registration number:**

NCT00241644

## Background

Rotavirus is the single most important cause of severe acute gastroenteritis worldwide in children under the age of five. The World Health Organization (WHO) estimates that rotavirus is associated with approximately 527,000 deaths globally, the majority of which (>85%) occur in young children in the developing countries of Asia and Africa [1,2]. Recently, WHO made a global recommendation for rotavirus immunization in all infants [3] based on the efficacy data generated in developing countries with both commercial rotavirus vaccines namely, *Rotarix*™ (GlaxoSmithKline Biologicals) and *Rotateq*™ (Merck and Co., Inc) [4-6].

In the pivotal efficacy studies conducted with these two rotavirus vaccines across Europe, Latin America, Asia and the USA, efficacy (ranging between 85% to 98%) was demonstrated against severe rotavirus gastroenteritis [7,8]. Additional clinical trials have confirmed the efficacy of the monovalent G1P[8] human rotavirus vaccine, *Rotarix*™ (GSK Biologicals, Rixensart, Belgium), against multiple rotavirus strains found to occur commonly in human infants [4,9-11]. A pooled analysis of the clinical studies performed with *Rotarix™* also demonstrated heterotypic protection of this vaccine against various human rotaviruses [12]. Nevertheless, uncertainty remains regarding the extent of cross protection provided by *Rotarix™*, particularly against strains that bear neither the G1 nor the P[8] antigens.

Rotaviruses are double stranded RNA (dsRNA) viruses composed of an outer capsid, an inner capsid and a core that houses the 11 segments of dsRNA. The viruses carry two neutralization antigens located on the outer capsid, which are known to elicit the production of serotype-specific neutralizing immune response in the host, and are considered important in vaccine development [13]. The outer capsid antigens – VP4 (P-type) and VP7 (G-type) - are categorized into various “genotypes” based on the molecular characterization of the genes encoding these two outer capsid proteins [14].

Among rotavirus strains, many combinations of the G- and P-types are possible, although a limited number have been commonly identified among human rotaviruses. So, G1P[8] is the most common strain circulating globally representing more than 50% of all human rotavirus strains, and G3P[8], G4P[8], G9P[8] and G2P[4] also occur commonly [14]. G2P[4] rotaviruses are distinct to the monovalent vaccine on both antigens, and have garnered special interest. These strains seem to appear in a cyclic nature in the human population, emerging as the dominant strain every 3–4 years [15,16].

Nevertheless, rotavirus strain diversity remains a complex issue for a number of reasons. Firstly, there are well-recognized geographic differences in the distribution and circulation of wild-type rotaviruses. G8 strains have had a peculiar predilection for Africa and occur much more frequently here than in other regions [17-21]; similarly G5 strains circulated widely in Latin America [14,22] and G10 strains were more common in India [23]. Secondly, new strains emerge through natural molecular evolution to appear in the human population, as demonstrated by the recent appearance of G12 strains [24,25]. Finally, strains also evolve through small “antigenic drift” changes in one of the outer capsid genes, thus eluding typing by the reverse-transcription polymerase chain reaction (RT-PCR) primers [18,26,27]. The VP4 types show similar diversity, although only three P-types (P[4], P[6] and P[8]) are common in human rotaviruses. In Africa, the P[6] type is identified much more commonly and can represent more than 50% of strains from symptomatic infections [28,29].

Epidemiological data have shown that Africa harbors a diverse range of rotavirus types, from the most common G1 type to the unusual G8, G9, G10 and G12 [28-30] types. In a large, randomized controlled trial conducted in Malawi and South Africa, *Rotarix™* was 61.2% efficacious in protecting infants from severe rotavirus gastroenteritis due to this wide range of diverse strains [4].

In the current paper we describe the rotavirus types identified in the phase III African trial and the efficacy of the monovalent G1P[8] human rotavirus vaccine in preventing severe rotavirus gastroenteritis caused by various circulating G- and P-rotavirus types.

## Methods

### Study design and participants

A double-blind, randomized, placebo-controlled, multi-center trial conducted in South Africa and Malawi enrolled healthy infants aged 5−10 weeks. Infants were randomly allocated into three groups (1:1:1) to receive either two doses of the *Rotarix™* vaccine at 10 and 14 weeks of age (two-dose group), three doses of *Rotarix™* at 6, 10 and 14 weeks of age (three-dose group) or three doses of placebo at 6, 10 and 14 weeks of age (placebo group). Further details of the study design have been presented elsewhere [4]. The study was conducted in accordance with Good Clinical Practice Guidelines. The study protocol, including the informed consent form was approved by the ethics committee of the World Health Organization and the ethics committee of the study centers. Informed consent was obtained for all participants prior to the start of study-related activities.

### Vaccine

The study vaccine *Rotarix™*, calcium carbonate buffer, and the placebo were developed and manufactured by GlaxoSmithKline Biologicals. The composition of the vaccine was the same as the commercial formulation, and the placebo was the same formulation without the viral antigen [31].

### Assessment of efficacy

Efficacy assessment was performed from 2 weeks after receipt of the third vaccine or placebo dose until one year of age. Infants were actively followed up by weekly visits to their homes to capture any gastroenteritis episodes during the study period. Gastroenteritis was defined as diarrhea with or without vomiting and diarrhea was defined as passage of three or more, looser than normal stools within a 24-hour period. Parents/guardians were instructed to collect stool samples during any gastroenteritis episode from Dose 1 of *Rotarix™* vaccine/placebo until one year of age. They were also advised to complete diary cards (e.g. number and duration of diarrhea and vomiting episodes, fever, dehydration and treatment administered) for each gastroenteritis episode. Gastroenteritis episodes were classified as two separate episodes if there was an interval of five or more symptom-free days between the episodes. Based on the information in the diary cards, the severity of each gastroenteritis episode was assessed using a 20-point Vesikari scale [32]. A score of ≥ 11 points indicated severe gastroenteritis.

Collected stool samples were tested for the presence of rotavirus antigen using an enzyme-linked immunosorbent assay (ELISA; RotaClone, Meridian Bioscience). All rotavirus positive stool samples were examined further with the use of a reverse-transcriptase-polymerase-chain-reaction (RT-PCR), followed by a reverse hybridization assay to determine the G and P types and in some cases, direct sequencing analysis of VP7 and VP4 PCR fragments was performed to confirm the G and P genotypes [33].

### Statistical analyses

All statistical analyses were performed using SAS 9.1 and 95% confidence interval (CI) calculated using Proc StatXact-7.

The follow-up period for efficacy was from 2 weeks after the last *Rotarix™* vaccine/placebo dose until one year of age. For the efficacy analysis, infants who had completed the full vaccination course, had entered the efficacy surveillance period (2 weeks after the last dose until one year of age) and had no wild-type (non-vaccine strain) rotavirus detected in their stool sample between Dose 1 and 2 weeks after last dose were included.

The study was powered only to observe a difference between pooled *Rotarix™* group and placebo group. Hence, for the efficacy analysis, a comparison between the pooled *Rotarix™* group and the placebo group was undertaken. The primary and secondary efficacy endpoint and the secondary safety and immunogenicity endpoints have been presented elsewhere [4].

In the present paper, the 95% CI for the percentage of infants with severe rotavirus gastroenteritis caused by circulating G and P types from 2 weeks post-last dose until one year of age was calculated for the pooled *Rotarix™* and the placebo groups (overall and per country). Respective vaccine efficacy was also calculated for each G- and P- type of virus with 95% CI. A two-sided Fisher exact test was used to calculate the p-value between groups; p-values < 0.05 were considered statistically significant.

## Results

### Study population and demography

A total of 4939 infants were individually randomly allocated into the three treatment arms (total vaccinated cohort); 1647 in the two-dose group, 1651 in the three-dose group (i.e. 3298 in the pooled *Rotarix™* group) and 1641 in the placebo group. Of the 4939 infants, 4417 were included in the primary efficacy analysis. The allocation of infants in each group and the reasons for withdrawal at each stage of the study has been presented earlier [4]. The major reasons for withdrawal from the efficacy analysis included loss to follow-up or out-migration from the study area, failure to receive the full course of vaccines / placebo, or withdrawal of informed consent [4].

The first dose of *Rotarix™* was given at 6.4 weeks (standard deviation [SD]: 0.98 weeks) in the 3-dose group and at 11.2 weeks (SD: 1.22 weeks) in the 2-dose group (placebo was given at the 6 week visit). The proportion of male and female infants was similar in both groups and ≥97.1% of infants belonged to the African race.

Vaccine efficacy against the primary outcome of severe rotavirus gastroenteritis in the ATP pooled rotavirus groups compared to placebo was 61.2% (95% CI: 44.0–73.2). The vaccine showed efficacy against severe rotavirus gastroenteritis in both the 2-dose regimen (58.7%; 95% CI: 35.7–74.0) and the 3-dose regimen (63.7%; 95% CI: 42.4–77.8) as described in the original report [4].

### Common circulating rotavirus types and efficacy

**Figure 1:**
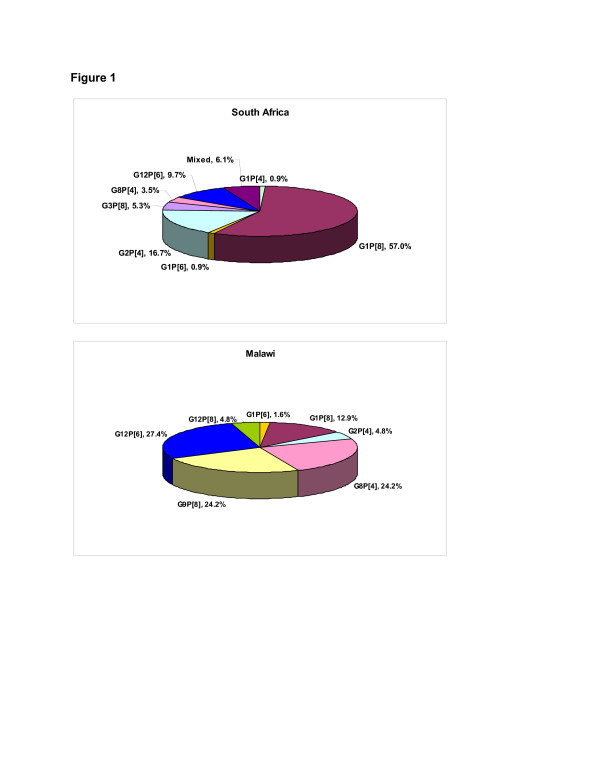
**Distribution of major rotavirus strains in placebo groups for South Africa and Malawi.** Figure obtained from Supplement to: Madhi SA, Cunliffe NA, Steele D, et al. Effect of human rotavirus vaccine on severe diarrhea in African infants. N Engl J Med 2010;362:289-98.

Diverse wild-type rotavirus types circulated during the study period including large numbers of G2, G8 and G12 types, with different distribution per country (Figure 1). G1 wild-type was the most predominant G type detected from 23 (1.6% [95% CI: 1.0%; 2.4%]) severe rotavirus gastroenteritis episodes in the placebo group. The other frequently circulating G types were G12 and G8, isolated from 15 (1% [95% CI: 0.6%; 1.7%]) severe rotavirus gastroenteritis episodes each in placebo group, respectively (Table 1).

**Table 1:** **Efficacy of*Rotarix™*against severe rotavirus gastroenteritis by rotavirus G-types in South Africa and Malawi (according to protocol cohort for efficacy)**

		***Rotarix™* pooled group**		**Placebo**		**Vaccine efficacy% (95% CI)**	**p-value**
		**N (n)**	**% (95%CI)**	**N (n)**	**% (95%CI)**		
Overall efficacy		2974 (56)	1.9 (1.4; 2.4)	1443 (70)	4.9 (3.8; 6.1)	61.2 (44.0; 73.2)	<0.001
Rotavirus type							
G1 wild-type	Overall	2974 (17)	0.6 (0.3; 0.9)	1443 (23)	1.6 (1.0; 2.4)	64.1 (29.9; 82.0)	0.002
	South Africa	1944 (11)	0.6 (0.3; 1.0)	960 (18)	1.9 (1.1; 2.9)	69.8 (32.5; 87.1)	0.0022
	Malawi	1030 (6)	0.6 (0.2; 1.3)	483 (5)	1.0 (0.3; 2.4)	43.7 (−133.1; 85.7)	0.3427
G2	Overall	2974 (3)	0.1 (0.0; 0.3)	1443 (7)	0.5 (0.2; 1.0)	79.2 (8.9; 96.5)	0.017
	South Africa	1944 (1)	0.1 (0.0; 0.3)	960 (6)	0.6 (0.2; 1.4)	91.8 (32.2; 99.8)	0.0065
	Malawi	1030 (2)	0.2 (0.0; 0.7)	483 (1)	0.2 (0.0; 1.1)	6.2 (−5433.1; 95.1)	1.0000
G3	Overall	2974 (2)	0.1 (0.0; 0.2)	1443 (6)	0.4 (0.2; 0.9)	83.8 (9.6; 98.4)	0.018
	South Africa	1944 (2)	0.1 (0.0; 0.4)	960 (6)	0.6 (0.2; 1.4)	83.5 (7.9; 98.4)	0.0187
	Malawi	1030 (0)	0.0 (0.0; 0.4)	483 (0)	0.0 (0.0; 0.8)	–	–
G8	Overall	2974 (11)	0.4 (0.2; 0.7)	1443 (15)	1.0 (0.6; 1.7)	64.4 (17.1; 85.2)	0.010
	South Africa	1944 (0)	0.0 (0.0; 0.2)	960 (5)	0.5 (0.2; 1.2)	100.0 (46.1; 100.0)	0.0039
	Malawi	1030 (11)	1.1 (0.5; 1.9)	483 (10)	2.1 (1.0; 3.8)	48.4 (−35.5; 80.1)	0.1553
G9	Overall	2974 (8)	0.3 (0.1; 0.5)	1443 (9)	0.6 (0.3; 1.2)	56.9 (−25.9; 85.5)	0.116
	South Africa	1944 (0)	0.0 (0.0;, 0.2)	960 (0)	0.0 (0.0; 0.4)	–	–
	Malawi	1030 (8)	0.8 (0.3; 1.5)	483 (9)	1.9 (0.9; 3.5)	58.3 (−21.7; 86.0)	0.0702
G12	Overall	2974 (15)	0.5 (0.3; 0.8)	1443 (15)	1.0 (0.6; 1.7)	51.5 (−6.5; 77.9)	0.051
	South Africa	1944 (1)	0.1 (0.0; 0.3)	960 (2)	0.2 (0.0; 0.8)	75.3 (−374.3; 99.6)	0.2555
	Malawi	1030 (14)	1.4 (0.7; 2.3)	483 (13)	2.7 (1.4; 4.6)	49.5 (−16.7; 78.0)	0.0933
Non-G1	Overall	2974 (39)	1.3 (0.9; 1.8)	1443 (47)	3.3 (2.4; 4.3)	59.7 (37.1; 74.4)	<0.001
	South Africa	1944 (4)	0.2 (0.1; 0.5)	960 (14)	1.5 (0.8; 2.4)	85.9 (55.1; 96.6)	0.0001
	Malawi	1030 (35)	3.4 (2.4; 4.7)	483 (33)	6.8 (4.7; 9.5)	50.3 (17.4; 70.0)	0.0048

N = number of infants included in each group.

n = number of infants reporting at least one event in each group.

% = percentage of infants reporting at least one event.

95% CI = 95% Confidence interval.

P-value = Two-sided Fisher Exact test; p-values < 0.05 indicate statistically significantdifference.

Vaccine efficacy against severe rotavirus gastroenteritis episodes caused by circulating G1, G12 and G8 rotavirus types was 64.1% (95% CI: 29.9%; 82%), 51.5% (95% CI:-6.5%; 77.9%) and 64.4% (95% CI: 17.1%; 85.2%), respectively (Table 1).

With respect to P-type, P[8] was dominant during the study period and was detected in 38 (2.6% [95% CI: 1.9%; 3.6%]) severe rotavirus gastroenteritis episodes in the placebo group; P[6] which has been described as a common VP4 type in Africa was only detected in 13 cases in placebo group (0.9% [95%CI: 0.5%; 1.5%] (Table 2).

**Table 2:** **Efficacy of*Rotarix™*against severe rotavirus gastroenteritis by rotavirus P-types in South Africa and Malawi (according to protocol cohort for efficacy)**

		***Rotarix™*pooled group**		**Placebo**		**Vaccine efficacy% (95% CI)**	**p-value**
		**N (n)**	**% (95%CI)**	**N (n)**	**% (95%CI)**		
Overall efficacy		2974 (56)	1.9 (1.4; 2.4)	1443 (70)	4.9 (3.8; 6.1)	61.2 (44.0; 73.2)	<0.001
Rotavirus type							
P4	Overall	2974 (12)	0.4 (0.2; 0.7)	1443 (20)	1.4 (0.8; 2.1)	70.9 (37.5; 87.0)	<0.001
	South Africa	1944 (1)	0.1 (0.0; 0.3)	960 (9)	0.9 (0.4; 1.8)	94.5 (60.4; 99.9)	0.0003
	Malawi	1030 (11)	1.1 (0.5; 1.9)	483 (11)	2.3 (1.1; 4.0)	53.1 (−19.3; 81.6)	0.1036
P6	Overall	2974 (12)	0.4 (0.2; 0.7)	1443 (13)	0.9 (0.5; 1.5)	55.2 (−6.5; 81.3)	0.052
	South Africa	1944 (1)	0.1 (0.0; 0.3)	960 (2)	0.2 (0.0; 0.8)	75.3 (−374.3; 99.6)	0.2555
	Malawi	1030 (11)	1.1 (0.5; 1.9)	483 (11)	2.3 (1.1; 4.0)	53.1 (−19.3; 81.6)	0.1036
P8	Overall	2974 (32)	1.1 (0.7; 1.5)	1443 (38)	2.6 (1.9; 3.6)	59.1 (32.8; 75.3)	<0.001
	South Africa	1944 (13)	0.7 (0.4; 1.1)	960 (22)	2.3 (1.4;, 3.4)	70.8 (39.5; 86.5)	0.0004
	Malawi	1030 (19)	1.8 (1.1; 2.9)	483 (16)	3.3 (1.9; 5.3)	44.3 (−15.8; 72.9)	0.0973

N = number of infants included in each group.

n = number of infants reporting at least one event in each group.

% = percentage of infants reporting at least one event.

95% CI = 95% Confidence interval.

P-value = Two-sided Fisher Exact test; p-values < 0.05 indicate statistically significant difference.

Vaccine efficacy against the respective P-types were 59.1% (95% CI: 32.8%; 75.3%) against P[8] and 55.2% (95% CI: -6.5%; 81.3%) against P[6] (Table 2). Interestingly, vaccine efficacy against the P[4] was 70.9% (95% CI: 37.5; 87.0).

### Country-specific results

G1 wild-type was the most common G type circulating in South Africa, and was detected in 18 (1.9% [95% CI: 1.1%; 2.9%]) severe rotavirus gastroenteritis episodes from placebo group. While in Malawi, G12 was the most commonly circulating G type isolated from 13 (2.7% [95% CI: 1.4%; 4.6%]) severe rotavirus gastroenteritis episodes from placebo group. Vaccine efficacy against G1 wild-type in South Africa was 69.8% (95% CI: 32.5%; 87.1%) and against G12 in Malawi was 49.5% (95% CI: -16.7%; 78.0%) (Table 1).

However, in both countries P[8] was the most frequently circulating P type, isolated from 22 (2.3% [95% CI: 1.4%; 3.4%]) severe rotavirus gastroenteritis episodes in South Africa and 16 (3.3% [95% CI: 1.9%; 5.3%]) episodes in Malawi in the placebo group. Vaccine efficacy against P[8] was 70.8% (95% CI: 39.5%; 86.5%) and 44.3% (95% CI: -15.8%; 72.9%) in South Africa and Malawi, respectively (Table 2).

## Discussion

Africa presents unique challenges for rotavirus immunization. First, the continent carries the highest burden of rotavirus mortality, where 12 of the 13 countries with greatest mortality rates per capita are located [2,34], and more than 250,000 children perish annually due to rotavirus [29,35]. Rotavirus vaccines are urgently needed in this region which would make a substantial contribution in reducing childhood deaths and hospitalizations due to rotavirus [36]. Most GAVI (Global Alliance for Vaccines and Immunization)-eligible countries are concentrated in Africa and the lowest global immunization coverage is also recorded here [37]. Given the high burden of rotavirus disease in Africa, the WHO recommends the early administration of rotavirus vaccines with the first two immunizations at 6 and 10 weeks of age [1].

Secondly, rotavirus strain diversity is extremely high in Africa with some novel G- and P-types circulating commonly [17,27-30]. Besides the globally emerging novel rotavirus strains, G9 and G12, which also occur commonly in Africa [21,24,25,38,39], G8 strains are frequently identified and seem to have an unusual affinity for Africa [17-21]. Furthermore, strains with the P[6] genotype circulate commonly in young African children with symptomatic rotavirus infection [17,28].

The wide circulation of diverse and unusual rotavirus strains in the region, emphasizes the importance of demonstrating cross-protective efficacy of the monovalent rotavirus vaccine, *Rotarix™* in preventing severe gastroenteritis [24,28,40]. Previous study has demonstrated that heterotypic protection may be due to the expression of serologically or genotypically identical proteins other than those encoded by the different G-types [41]. The immune response to the VP4 antigen has been demonstrated to be significant [42], and there are cross-reactive epitopes on the VP4 protein [43]. The relative lack of diversity among P-types [42] when compared with the G types, may aid in heterotypic protection as suggested previously [43]. In addition, protection may be offered via immune effector mechanisms other than neutralizing antibody [44].

In the present paper, in addition to the common G1 and P[8] types, we observed five G types (G2, G3, G8, G9 and G12) and two P types (P[4] and P[6]) in circulation during the study period. Importantly, the G8 and G12 types have not been observed in earlier efficacy studies providing the opportunity to assess vaccine efficacy against these novel types [9-11]. Similarly, the numbers of strains bearing the P[4] genotype with various G-types, all heterotypic to the G1P[8] vaccine strain, enable an assessment of vaccine efficacy against truly heterotypic strains. The strain combinations used to generate the results include 8 G2P[4] strains, 19 G8P[4] strains and a single G8P[6], and 23 strains bearing G12P[6] specificity.

The overall vaccine efficacy of the monovalent rotavirus vaccine in preventing severe rotavirus gastroenteritis in African infants was previously reported as 61.2% (95% CI: 44%; 73.2%) [4]. G1 wild-type was the predominant circulating rotavirus type isolated from 23 severe rotavirus gastroenteritis episodes in placebo group. Interestingly, the pattern of circulation of rotavirus types differed considerably between South Africa and Malawi during the study period. Unlike South Africa, where G1 was predominantly circulating (isolated from 18 severe rotavirus gastroenteritis episodes in placebo group) similar to worldwide epidemiology, this was not the case in Malawi, where G1 wild-type strains were the lowest seen in more than a decade of surveillance [45]. In Malawi, G12 was the predominant rotavirus type (isolated from 13 severe rotavirus gastroenteritis episodes in placebo group), as observed in an earlier study by Cunliffe et al, where G12 was identified as a newly emerging rotavirus type in Malawi [24]. Furthermore, G9 was circulating only in Malawi during the study period and hence the overall efficacy data on G9 rotavirus type reflected the Malawi-specific situation.

We can anticipate that the monovalent rotavirus vaccine will provide protection against the circulating rotavirus types that shared either the G or the P type with the vaccine strain (homotypic protection). However, the G2 and G8 types were all circulating in combination with P[4] type (with a single strain bearing G8P[6] specificity), sharing neither the G or the P type with the vaccine strain. It is therefore important to note that significant protection was afforded by the vaccine against severe gastroenteritis caused by these dually heterotypic rotavirus types (vaccine efficacy against G2: 79.2% [95% CI: 8.9%; 96.5%; p -value = 0.017]; vaccine efficacy against G8: 64.4% [95% CI: 17.1%; 85.2%; p-value 0.010]; vaccine efficacy against P[4]: 70.9% [95% CI: 37.5%; 87.0%]). This is an important observation as the earlier efficacy studies showed limited heterotypic protection [9-11], and there has been some suggestion that the monovalent vaccine may not confer cross protection against non-vaccine strains.

These data are encouraging because with the diversity of the rotavirus types in circulation and the global emergence of new strains in the human population, homotypic protection alone will be unlikely to provide complete protection against severe rotavirus gastroenteritis. Heterotypic protection of the rotavirus vaccine is important to effectively reduce the rotavirus disease burden.

## Conclusions

The high burden of rotavirus disease and mortality in Africa, coupled with the great diversity and distribution of rotavirus strains differing from year-to-year and region-to-region within the African continent show the clear need for an effective and safe vaccine, which is able to offer heterotypic protection against multiple strains. In this study, *Rotarix™* vaccine demonstrated efficacy against severe gastroenteritis caused by diverse circulating rotavirus types, including rotaviruses sharing neither G nor P type with the vaccine strain.

Rotavirus surveillance efforts are needed in Africa to elucidate the burden of disease and the strain diversity in the region; but importantly to provide a platform against which the impact of the vaccines can be assessed once they are introduced. Rotavirus surveillance after the introduction of routine vaccination could further explore the concept of heterotypic protection in a real-life setting.

## Abbreviations

ATP: According to protocol; CI: Confidence interval; dsRNA: Double-stranded RNA; ELISA: Enzyme-Linked Immunosorbent Assay; GSK: GlaxoSmithKline; RT-PCT: Reverse-Transcriptase Polymerase Chain Reaction; SAS: Statistical analysis system; SD: Standard deviation.

## Competing interest

Dr. Madhi reports receiving lecture and consulting fees from GlaxoSmithKline and consulting fees from Merck; Dr. Cunliffe, receiving consulting fees and grant support from Sanofi Pasteur and GlaxoSmithKline and Suryakiran and Dr. Han are employees of GlaxoSmithKline; Dr. Han owns shares in GlaxoSmithKline. No other potential conflict of interest relevant to this article was reported.

## Authors’ contribution

ADS: PI, overall study design, data review, interpretation of results and review, drafting of manuscript, approval of study report, HHH: study design, overall management, data review, interpretation of results and review and approval of study report, CL: protocol design, supervision and management of study implementation in South Africa, training of investigators, 486 subjects contributed to the study, review of various publications related to this study, SA: protocol design, supervision and management of study implementation in South Africa, training of investigators, training of investigators, review of the clinical study report, review of various publications related to this study, LJVD: Design and development of the method, development and validation of the testing algorithm, supplemental (sequence) analysis in samples with aberrant results. All authors read and approved the final manuscript.

## Pre-publication history

The pre-publication history for this paper can be accessed here:

http://www.biomedcentral.com/1471-2334/12/213/prepub
